# Traditional and Indigenous Fruits and Vegetables for Food System Transformation

**DOI:** 10.1093/cdn/nzab092

**Published:** 2021-07-06

**Authors:** Gina Kennedy, Rebecca Kanter, Sinee Chotiboriboon, Namukolo Covic, Treena Delormier, Thingnganing Longvah, Patrick Maundu, Nasrin Omidvar, Prakash Vish, Harriet Kuhnlein

**Affiliations:** Chair, International Union of Nutritional Sciences (IUNS) Task Force on Traditional and Indigenous Food Systems and Nutrition, Falls Church, VA, USA; Department of Nutrition, Faculty of Medicine, University of Chile, Santiago, Chile; Institute of Nutrition, Mahidol University, Salaya, Thailand; International Food Policy Research Institute, Addis Ababa, Ethiopia; Centre for Indigenous Peoples’ Nutrition and Environment and School of Human Nutrition, McGill University, Montreal, Canada; National Institute of Nutrition, Hyderabad, India; Kenya Resource Centre for Indigenous Knowledge, National Museums of Kenya, Nairobi, Kenya; Department of Community Nutrition, National Nutrition and Food Technology Research Institute, Faculty of Nutrition Sciences and Food Technology, Shahid Beheshti University of Medical Sciences, Tehran, Iran; Ramaiah University of Applied Sciences, Bangalore, India; Centre for Indigenous Peoples’ Nutrition and Environment and School of Human Nutrition, McGill University, Montreal, Canada

**Keywords:** fruits, vegetables, indigenous, traditional, diet

## Abstract

Fruit and vegetable consumption is recommended in numerous food-based dietary guidelines and forms a key recommendation in many international statements related to healthy diets. There are thousands of fruit and vegetable species from which to choose, but despite this abundance from nature, populations in most countries neither produce nor consume the recommended daily amounts of fruits and vegetables. There is enormous potential to better incorporate the wealth of diverse fruit and vegetable species and varieties into food systems. Known and preserved by indigenous communities, these hidden food treasures can foster collaborative research and learning. This perspective from the Task Force on Traditional and Indigenous Food Systems and Nutrition of the International Union of Nutritional Sciences (IUNS) highlights 5 key actions that can be taken by individuals, communities, and nations to reshape dialogue about traditional and indigenous fruits and vegetables to benefit people and planetary ecosystems.

## Introduction

The importance of a diversity of fruits and vegetables in human diets to maintain good health is well established. In 2003, the World Health Organization and the Food and Agriculture Organization recommended a population intake goal of 400 g/d or more of fruits and vegetables, citing that since the nutritional benefits cannot be ascribed to a single or mix of nutrients and bioactive substances the recommendation is for consuming a minimum quantity of these foods (1). The 2019 EAT Lancet Report includes healthy diet reference targets for a 2500 kcal diet of 300 g of vegetables and 200 g of fruit (2). A 2019 review of food-based dietary guidelines (FBDG) from all 90 countries in which FBDG are available indicated that 84 out of 90 guidelines provide ≥1 and sometimes several specific message(s) on fruits and vegetables (3). The most common messages related to fruits and vegetables were “Eat Daily” and “Eat a Variety” and about a fifth of the guidelines suggested “Eat More.”

Despite this compelling public policy advice, at population level nearly all countries are falling below the recommended intakes for fruits and vegetables (4). The average global intake of vegetables is reported as 209 g/d and for fruits is 81 g/d,  with wide variation across and within countries (5). There are many reasons for this divergence from public health recommendations. The most commonly cited barriers are availability, accessibility, affordability, and desirability (6). The production of fruits and vegetables is, on average, 22% below levels needed to meet consumption requirements (7). However, thousands of fruit and vegetable species are available that could be produced and promoted to improve intakes (8). A 2018 review of vegetable species documented the existence of 1097 edible species in the world, most of which are underutilized in current food systems (9). This number of edible species represents only a fraction of fruit and vegetable diversity as many species include hundreds of varieties and cultivars (8). There is also political commitment with the UN Sustainable Development Goals (SDGs): under SDG 2, target 5, to maintain genetic diversity and cultivate plants and wild relatives (10).

Although Indigenous Peoples’ collective experience contains knowledge from 22% of the world's ecosystems and the majority of the planet's biodiversity, their poor diet quality and health disparities are well-documented (11). There are significant sociocultural barriers to increasing intake of fruits and vegetables not only by Indigenous Peoples, but also by populations at large. Indigenous Peoples face colonization and lack of support from government to maintain the interconnections of cultural identity and environmental productivity resulting in loss of their food resources, health, and well-being (12).  For people living in poverty, fruits and vegetables are often not a top priority in purchasing patterns, where the first priority is to alleviate hunger. More costly sources of dietary diversity, which often include fruits and vegetables are considered unaffordable (6).

Given the body of agreement in dietary guidelines on the health benefits of fruit and vegetable consumption, as well as the high level of global and national policy congruence, it is striking that per capita intake of fruits and vegetables has not increased. There remains enormous potential to tap into the wealth of biodiverse fruit and vegetable options within traditional and indigenous food systems. Many of the species and varieties contain high nutritional value, have low water requirements, are adapted to poor quality soils, and demonstrate good resistance to pests and diseases (9). Many are adapted to grow in diverse and contrasting climates, some are tolerant to cold and others to heat; some are drought tolerant whereas others are flood resistant. To date, traditional and indigenous food systems continue to be disconnected from “mainstream” food systems and food culture to the detriment of current diets and sustainability of food systems globally.

## Five Key Interconnected Actions Recommended by the IUNS Task Force on Traditional and Indigenous Food Systems and Nutrition to Ignite Action to Increase Consumption of Traditional Fruits and Vegetables

### Words matter

Traditional and indigenous foods need to be carefully described and categorized. “Traditional foods” can originate from any culture. Kuhnlein et al. propose a definition for a traditional food system as: “all food from a particular culture available from local resources and culturally accepted. It includes sociocultural meanings, acquisition/processing techniques, use, composition, and nutritional consequences for people using the food” (p.19) (13). “Traditional food” is a generic term that can be applied to any family, community, culture, nation, or region (for example, “her” family's traditional food, Anacortes town's traditional food, Greek traditional food, northwest coast traditional food, etc.). The term “indigenous food” can refer to either a species or variety that is naturally occurring (indigenous) in a particular geographic place. For example, *Strepthanthella longirostris* (little twist flower) is an annual wild green vegetable that grows in sandy soils of the southwestern USA. It is called Homima in the Hopi language where it is harvested in early March on the Hopi reservation in Arizona and is considered an “Indigenous food” of the Hopi Indigenous People (14).

Indigenous Peoples are identified according to criteria described by the UN Permanent Forum on Indigenous Issues (15). They have characteristics clearly different from other segments of a nation's population, and their food system contains species, use, and cuisine defined with their cultural designation (for example, Nuxalk traditional food, or the indigenous food of the Nuxalk People). For Indigenous Peoples it is useful and respectful to recognize their cultural designation when referring to foods they frequently harvest and consume. There may be political nuance in the interchangeable use of these terms that reflects “ownership” context of the food resource. It is reasonable to assume that readers from the Americas, Europe, and Oceania are sensitive to the careful use of the designations of indigenous foods because of the lasting colonial history of indigenous territories and their original Indigenous Peoples.

Attention to use of correct and respectful nomenclature is often missing in descriptions of traditional or indigenous foods. Towns and Shakelton (2018) documented 76 terms used in current literature to describe African vegetables, terms that are sometimes used interchangeably (16). They propose a standardized definition of traditional African vegetables as “plant species that are indigenous or naturalized to Africa, well adapted to or selected for local conditions, whose plant parts are used as a vegetable and whose modes of cultivation, collection, preparation, and consumption are deeply embedded in local cuisine, culture, folklore, and language” (p.11) (16).

Terms such as “forgotten,” “neglected,” and “orphaned” are often used in the agriculture literature to describe traditional and indigenous species. The general perception of traditional foods as food for the poor or food for animals (e.g. hog plum, monkey bread) is often documented and has led to these foods being associated with social stigma (17). Examples of nonstigmatizing language such as “future smart food” and “hidden food treasures” need to be globally adopted and used (18).

### FBDG as a policy platform to encourage traditional and indigenous foods

FBDG are simple and practical translations of scientific, nutrient-based recommendations into total diet concepts meaningful to consumers. They are developed because people select foods, rather than individual nutrients and they serve to guide a range of policies and programs that help to inform agriculture, health, nutrition, and education sectors (19). FBDG represent a unique opportunity to reach multiple sectors and encourage positive action for healthy diets and sustainable food systems and give the opportunity to develop more regional- and culturally specific FBDG. It is expected that FBDG highlight local and traditional, as well as seasonally available foods by specific areas within a country or region. Although this aspect has been addressed in just a few FBDG, it could lead the way for other national guidelines. The FBDG of Brazil provides a good example by giving consumers information on region-specific diets and how to select healthy options for breakfast, lunch, and dinner (20).

Canada's FBDG provide another example of cultural specificity. The 2007 version of Canada's Food Guide has an adapted food guide for First Nation, Metis, and Inuit, which are the 3 federally recognized groups of Indigenous Peoples in Canada (21). The 4 food groups are presented in a circle and represent food items familiar to these groups, rather than on the rainbow motif used on the mainstream guide. In the center of the circle of food groups is an illustration depicting Indigenous Peoples’ food systems from people active in traditional food practices happening in relation to the land, water, and sky. The purpose of the food guide is to value the cultural practices and relation with the environment central to Indigenous Peoples’ food systems (22). Canada's evidence-based 2019 Dietary Guidelines emphasize the importance of healthy diets for all Canadians including Indigenous Peoples and refers to how their traditional foods can improve diet quality. Examples are given of traditional foods from the different regions and Indigenous cultures of Canada and explain how traditional local foods can be consumed safely (23). Attention to Indigenous cultures in the guidelines helps transform the political and social landscape of reconciliation in Canada that establishes and maintains a mutually respectful relationship between Indigenous and non-Indigenous peoples.

The development of new FBDG in countries such as Ethiopia represents a key platform for the recognition and valorization of local traditional foods within food systems (24).

### Culinary reawakening tied to culture

Promoting culinary habits or culinary reawakening tied to culture can valorize and increase use of traditional and indigenous fruits and vegetables. Although the nutritional benefits of dietary diversity related to traditional and indigenous species and foods seem to be undervalued by modern food systems, some in the culinary world embrace traditional and Indigenous food cultures. Slow Food, its Indigenous Terra Madre network, and the White Earth Land Recovery Project (USA), and others, promote indigenous knowledge from the grassroots level. New recipes within restaurants as well as many new products on the market, include traditional fruits and vegetables (25, 26). Worldwide, many restaurants strive to serve only local, seasonal produce on their menus; whereas others, for example the Sweet Green restaurant chain (www.sweetgreen.com), provide consumers the name and location of the farm where the produce has been sourced. Media documentation of traditional and indigenous foods and food systems is becoming more common and part of popular culture. The popular Red Chef Revival docuseries that follows 3 Indigenous Chefs, explores modern indigenous cuisine from their unique perspectives (27). This is just one of many examples of food-based docuseries celebrating indigenous and traditional foods and culture available on modern media (television, YouTube).

### Raise public awareness of the diversity of fruits and vegetables

Food fairs, cooking competitions, farmers’ markets, and local community cooking demonstrations can all be used to raise awareness of the benefits and role of traditional and indigenous fruits and vegetables in local food systems. In Italy, for example, *sagra* (local food festivals) are traditional means of celebrating seasonally available local produce and also serve to strengthen cohesion within the community (28). The *sagra* take place during the seasonal peak of availability and in specific geographic locations of the celebrated local food. There are *sagra* in Italy nearly every week of the year with diverse traditional foods and multiple varieties of for example grapes, persimmon, pomegranate, and chestnuts that are valorized and recognized for their unique role in Italian food culture.

In Hyderabad, India, the Deccan Development Society works within the Alternative Public Distribution System and conducts many activities promoting local foods, including food festivals, fostering sustainable food processing equipment, recipe competitions, developing organic farming cooperatives, and production of films stressing local foods. Research showed improved nutrient intakes of children in the associated villages (12).

Other examples include the Farm to School Grant Program of the USDA that provides funds and evaluation of programs that foster sourcing of local foods for school districts (29). Another excellent example of a school-based program is the Edible Schoolyard Project in Berkeley, California, that is supported with education activities from an established local restaurant and celebrated chef, Alice Waters (30). Dietary improvement with micronutrient-rich fruit was successful with a program in Pohnpei, Micronesia, that stressed social marketing that in turn created awareness that developed the demand that led to increased local production (31).

### Incorporate traditional and indigenous foods and food systems into research

More research is needed to describe how food systems research can lead to understanding and inclusion of more traditional and indigenous foods, particularly fruits and vegetables. Lack of funding and research for work on traditional and indigenous foods runs the gamut from the collection of the essential data on food species identifications and composition, as well as basic production and dietary intake statistics, to more context-specific research on understudied value chains, agricultural production systems, and associated market opportunities, as well as variation in production, preservation, and consumption by culture. Given the global need for greater attention to fruit and vegetable availability, access, affordability, and acceptability, all options and opportunities for increasing data on fruits and vegetables should be explored. The unlimited variety of global fruits and vegetables are underutilized resources that provides great opportunity to augment public information and increase demand and subsequent supply of these healthy foods.

Indigenous Peoples’ food systems are often overlooked, understudied, and undervalued, yet many traditional food species could be revalorized for a more colorful plate and wider selection to match consumer taste and preference. Research on Indigenous Peoples’ food systems can reveal a wealth of new knowledge about promising fruits and vegetables provided that the people directly participating in this knowledge are full partners in shared benefits (32).

Documenting Indigenous Peoples’ food systems with data on species/varieties, parts, preparation techniques, nutrient composition, and taste appreciation provide data important for biodiversity conservation and nutrition promotion programs for Indigenous communities featuring fruits and vegetables (12, 13, 32). This can lead to health promotion programs that value the cultural and indigenous food knowledge central to the identity and cultural continuity of Indigenous Peoples.

Culturally appropriate and climate-adapted species have been used and maintained by Indigenous Peoples for generations, often millennia, in stable food systems. Traditional local foods and diets have shown lower water and carbon footprints in comparison to modern commercial foods (33). Promoting culture and local production to incorporate more fruits and vegetables in markets gives an opportunity for improved livelihoods among these people, often rural and poor, who know how to grow, preserve, and prepare these unique crops.

Most, if not all, global nutrition transitions occur in poor-quality diets that emphasize ultraprocessed foods that target low-income areas or low- and middle-income countries. This emphasizes the need to increase use of nutrient-dense fruits and vegetables in fixed energy/calorie budgets (34, 35). Reflected within both the rural and urban poor in many countries, all resident cultures need education and research-based support to maintain their healthy food traditions.

## Conclusion

Fruit and vegetable consumption is recommended by the majority of the world's FBDG, but consumption is well below suggested levels in most countries. In some areas, the production level of fruits and vegetables is below what is needed; despite the abundance of diverse types of traditional and indigenous fruits and vegetables that could be better utilized. The 5 key actions described in this perspective piece provide a way forward to improve the availability, affordability, and desirability of indigenous and traditional fruits and vegetables and engage in needed food systems transformation.

## Notes

The publication of this article was supported by the Shakopee Mdewakanton Sioux Community, through a gift to the University of Minnesota from its Seeds of Native Health campaign.

Author disclosures: The authors report no conflicts of interest.

Abbreviations used: FBDG, food-based dietary guidelines; IUNS, International Union of Nutritional Sciences; SDG, sustainable development goal.
